# Managing Hepatotoxicity Caused by Anti-tuberculosis Drugs: A Comparative Study of Approaches

**DOI:** 10.34172/aim.2024.19

**Published:** 2024-03-01

**Authors:** Faeze Abbaspour, Malihe Hasannezhad, Hossein Khalili, SeyedAhmad SeyedAlinaghi, Sirous Jafari

**Affiliations:** ^1^School of Medicine, Tehran University of Medical Sciences, Tehran, Iran; ^2^Iranian Research Center for HIV/AIDS, Iranian Institute for Reduction of High-Risk Behaviors, Tehran University of Medical Sciences, Tehran, Iran; ^3^Department of Infectious Diseases, Imam Khomeini Hospital Complex, Tehran University of Medical Sciences, Tehran, Iran; ^4^Department of Clinical Pharmacy (Pharmacotherapy), Faculty of Pharmacy, Tehran University of Medical Sciences, Tehran, Iran

**Keywords:** Anti-tuberculosis drugs, Anti-tuberculosis regimen, Drug-induced hepatotoxicity, Drug-induced liver injury, Rechallenge treatment, Tuberculosis

## Abstract

**Background:**

Tuberculosis (TB) is one of the oldest and most well-known diseases that has been associated with humans for many years and remains a global health challenge today. Timely diagnosis and proper treatment are crucial for controlling and preventing the spread of the disease. While anti-TB drugs offer many benefits, inadequate monitoring can lead to a range of side effects, including hepatotoxicity, which is a major concern and can cause treatment discontinuation. The aim of this study was to determine the approach to the hepatotoxicity of anti-TB drugs and to investigate potential relationships between demographic factors, underlying medical conditions, and successful retreatment outcomes for hepatotoxicity induced by anti-TB drugs.

**Methods:**

For this study, we reviewed the medical records of patients who experienced hepatotoxicity due to anti-TB treatment and were admitted to the infectious ward of Imam Khomeini Hospital between April 2015 and February 2019. The data were collected using a questionnaire.

**Results:**

The findings indicated that the female gender, weight loss at the beginning of hospitalization, *hepatitis C virus*, *hepatitis B virus (HBV)*, heart disease, and high levels of aspartate aminotransferase (AST) and alanine transaminase (ALT) at the beginning of hepatotoxicity are risk factors for failure to the retreatment of hepatotoxicity. There were two different approaches to the anti-TB retreatment regimen. The first approach involved gradually starting the drugs in full dose, while the second approach encompassed starting the drugs in the minimum dose and then increasing to the maximum dose. The results demonstrated no significant difference between the two approaches to managing hepatotoxicity induced by anti-TB drugs.

**Conclusion:**

Drug-induced hepatotoxicity is a common occurrence that often results in treatment discontinuation. Understanding the prevalence of this complication and identifying appropriate methods of rechallenge treatment is crucial to reducing complications and mortality rates.

## Introduction

 Tuberculosis (TB) remains a leading cause of serious illnesses in developing countries, currently ranking as the 13^th^ deadliest disease in the world. The prevalence of TB is significant, with more than one-third of the global population infected due to the Mycobacterium TB bacteria.^1^ While TB is a curable disease if diagnosed and treated properly, the use of anti-TB drugs can result in intense and unfavorable side effects, including hepatotoxicity.^2,3^ The combination of isoniazid (INH), rifampin (RIF), ethambutol (EMB), and pyrazinamide (PZA) is widely considered to be the most effective treatment for TB; however, some patients may not be able to tolerate these drugs and may not respond to the treatment.^4,5^ It should be noted that while many anti-TB drugs can cause hepatotoxicity, PZA, INH, and RIF are the main drugs that are known to cause complications.^6-9^

## Materials and Methods

 This study employed a cross-sectional, descriptive-analytical design to investigate the methods of the re-introduction of anti-TB drugs following the occurrence of hepatotoxicity during TB treatment. The study also sought to determine the preferred method of retreatment in the Department of Infectious Diseases at the Imam Khomeini Hospital Complex, where general patients with TB are referred. The census sampling method was utilized, and all the records of patients hospitalized in the department who experienced hepatotoxicity during the designated period and subsequently underwent re-treatment were extracted and underwent analysis.

 Drug-induced hepatotoxicity was defined as:

1. Elevation of serum aspartate aminotransferase (AST) or alanine transaminase (ALT) levels to greater than three times the upper limit of normal, in conjunction with the clinical symptoms of hepatitis.
2. Elevation of serum AST or ALT levels to greater than five times the upper limit of normal, with or without accompanying clinical symptoms.
3. Elevation of *alkaline phosphatase (*ALP) levels to greater than two times the upper limit of normal, in conjunction with symptoms such as jaundice and pruritus, or hyperbilirubinemia.^9^

Patients who voluntarily discontinued TB treatment before rechallenge, patients with unavailable regimen information, or those who referred to another clinic after reinitiating anti-TB drugs were excluded from the study.

 For patients who were diagnosed with drug-induced hepatotoxicity, after discontinuing all hepatotoxic drugs (INH, RIF, and PZA), alternative treatment options such as EMB, levofloxacin, and an aminoglycoside antibiotic were substituted in some cases. The anti-TB drugs, including INH, RIF, EMB, and PZA, were reintroduced once the serum transaminase levels had been normalized.

 Three re-initiation methods were used, including (1) full-dose administration of all anti-TB drugs from day 1 (INH/RIF/PZA), (2) full-dose administration of RIF (from day 1), INH (from day 8), and PZA (from day 15), and (3) a gradual dose escalation, starting with a minimum dose and increasing to the full dosage over time.^10^

 Additionally, a questionnaire was designed to collect relevant information (Table 1), including the patient’s demographic characteristics, duration of anti-TB treatment, mortality, type of drug regimen at the start of treatment, and any accompanying factors such as a history of liver disease, alcohol consumption, concurrent use of hepatotoxic drugs, and abnormal body mass index.

**Table 1:** Clinical Characteristics of Study Patients

**Parameters**	**Number (%) of Patients**
Total number of patients	52
Gender	
Male	36 (69.2)
Female	16 (30.8)
Age	
0‒20 years	1 (1.9)
21‒40 years	21 (40.3)
41‒60 years	18 (34.6)
> 60 years	12 (23)
Status of the viral infection	
HBV	1 (1.9)
HCV	13 (25)
HIV	23 (44.2)
History of smoking	33 (63.5)
History of alcohol consumption	8 (15.4)
History of drug abuse	29 (55.8)
Co-morbidity	
Cardiovascular diseases	8 (25)
Renal diseases	5 (15.6)
Pulmonary diseases	11 (34.3)
Rheumatologic diseases	3 (9.3)
Malignancies	5 (15.6)
Type of liver injury	
Hepatocellular	37 (71.2)
Cholestatic	3 (5.8)
Mixed hepatocellular- cholestatic	12 (23.1)
Clinical manifestations of hepatotoxicity	
Nausea/vomiting	8 (15.3)
Nausea/vomiting + abdominal pain	14 (26.9)
Jaundice	13 (25)
Asymptomatic	17 (32.6)

*Note*. HBV: *Hepatitis B virus*; HCV: *Hepatitis C virus*; HIV: Human immunodeficiency virus.

 The obtained data were analyzed using SPSS, version 26. Statistical tests, including the chi-square test, independent *t* test, and Pearson correlation, were applied to determine the level of significance. Drug-induced hepatotoxicity was considered the dependent variable, while all factors included in the questionnaire were considered independent variables. A *P* value of less than 0.05 was statistically significant. For the categorical variables, the authors chose to code variables as 1 = yes and 2 = no. As regards gender, it was considered 1 and 2 for female and male, respectively, and finally, for rechallenge regimen type, 1 and 2 represented “sequentially full dose” and “sequentially increased dose”, respectively. Regarding continuous variables, “liver function tests” and “hospitalizations” were scored by number based on units per liter (U/L) and days, respectively.

## Results

 In this study, 77 patients with TB were identified who suffered hepatotoxicity after taking anti-TB drugs. Since the information related to 25 patients was incomplete in the context of restarting drug treatment, they were excluded from the study, and finally, 52 patients were included in the study.

 According to Table 1, the study population consisted of 52 patients (69.2% male and 30.8% female), of whom 29 (55.8%) had a history of drug abuse, with 13 (44.8%) cases reporting intravenous drug use. Additionally, 33 (63.5%) patients had a history of smoking, and 8 (15.4%) had a history of alcohol consumption. One of the patients (1.9%) was diagnosed with hepatitis B, while 13 patients (25%) were found to have hepatitis C, and 23 patients (44.2%) were identified as having human immunodeficiency virus.

 Based on the serum levels of AST, ALT, and ALP, liver injury was classified into hepatocellular, cholestatic, and mixed hepatocellular-cholestatic categories. The most prevalent type of liver injury was hepatocellular, which was observed in 37 (71.2%) patients.

 In the study population of 52 patients, 17 (32.6%) were asymptomatic, 14 (26.9%) presented with vomiting and abdominal pain, 13 (25%) had jaundice, and 8 (15.3%) had vomiting as the only clinical symptom.

 In this study, none of the patients underwent the re-initiation of anti-TB treatment with all drugs at full dosage simultaneously. Instead, 21 (40.4%) patients had the anti-TB drugs reintroduced sequentially at full dosage, while 31 (59.6%) of them had the drugs introduced sequentially at a low dosage, which was gradually increased to the full dosage.

 In evaluating the type of anti-TB regimen that was initially administered to the patients, it was found that 33 (63.5%) received a three-drug regimen consisting of RIF, INH, and EMB. This three-drug regimen was the most commonly used in the reintroduction of treatment. Additionally, 16 (30.8%) patients received a four-drug regimen, including RIF, INH, EMB, and PZA. Further, 28 (53.8%) patients had one or more anti-TB drugs omitted in the re-administration regimen. The most common drug eliminated from the re-administration regimen was PZA, which was omitted in 25 (48%) patients.

 In this study, 48 (92.3%) patients tolerated the reintroduction treatment, while only 4 (7.7%) had an increase in liver enzymes (the recurrence of hepatotoxicity) following the reintroduction of PZA. Out of the total population, 42 (80.8%) were discharged from the hospital without any complications, 5 (9.6%) patients expired, and another 5 (9.6%) left the hospital with personal consent before the normalization of liver enzymes. The cause of death in all 5 patients was cardiopulmonary arrest, and none of the patients died due to liver complications. The mean duration of hospitalization for the patients was 25.83 days.

 The relationship between demographic variables, underlying diseases, and drugs used by patients with anti-TB re-challenge treatment tolerance (the recurrence of hepatotoxicity) was examined, and the results indicated a statistically significant relationship between *hepatitis C virus* (HCV) infection and anti-TB re-challenge treatment tolerance (*P*= 0.04, odds ratio [OR] = 11.4, 95% confidence interval [CI] = 1.06‒121.7). Moreover, there was a statistically significant association between weight loss and anti-TB re-challenge treatment tolerance (*P* = 0.01, OR = 9, 95% CI = 1.44‒176.3). The examination of the relationship between demographic variables, underlying diseases, and drugs used by patients with the duration of hospitalization revealed that patients with underlying heart disease (*P* = 0.02, Hedge’s g = 0.79, 95% CI = -18.68 ‒ -4.48) and *hepatitis B virus (*HBV) infection (*P* = 0.03, Hedge’s g = 0.06, 95% CI = 1.09‒30.9) had a statistically significant longer duration of hospitalization.

 An analysis provided the relationship between demographic variables, underlying diseases, and drugs used by patients with the improvement of hepatotoxicity after anti-TB treatment reintroduction. The results confirmed a statistically significant association between gender and recovery from liver injury (*P* = 0.03, OR = 4.8, 95% CI = 1.12‒20.47).

 The statistical indicators of the serum level of liver enzymes at the beginning and end of liver injury were presented, and the results represented that a higher level of AST (*P* = 0.005, Pearson r = 0.38, 95% CI = 0.12‒0.59) and ALT (*P*-value = 0.006, Pearson r = 0.37, 95% CI = 0.11‒0.37) enzymes at the onset of hepatotoxicity is a significant risk factor for an extended duration of hospitalization.

Table 2 examines the relationship between the type of anti-TB re-challenge regimen and the duration of hospitalization. The results (Table 2) indicated that there is no statistically significant association between the type of anti-TB re-challenge regimen and the duration of hospitalization. This suggests that the use of either method of reintroduction (sequentially reintroducing drugs at full dosage or gradually increasing dosage) does not significantly impact the outcome of treatment. However, the data demonstrated a statistically significant relationship between the removal of medication in the re-challenge regimen and the duration of hospitalization (*P* = 0.04, Hedge’s g = 0.57, 95% CI = 1.11‒4.12).

**Table 2:** Association Between Types of Rechallenge Regimen and Duration of Hospitalization

**Variable**		**Frequency**	**Standard Deviation**	**Average Days of Hospitalization**	***P*****-value**
Rechallenge regimen type	Sequentially full dose	21	15.10	26.14	> 0.9
	Sequentially increase dose	31	14.99	25.84	
Drug omitting	Yes	28	8.71	22.11	0.04
	No	24	19.06	30.46	

## Discussion

 The present study investigated and compared different methods of reintroduction of anti-TB drugs.

 In this study, the majority of patients diagnosed with anti-TB-induced hepatotoxicity were more than 40 years old, although there was no statistically significant relationship between older age and the poor outcome of hepatotoxicity treatment. In some studies, an age-related decline in the liver’s ability to metabolize xenobiotics has been proposed as a risk factor for anti-TB drug-induced hepatotoxicity.^11-13^ In previous studies conducted in Iranian populations, advanced age has not been identified as a risk factor for anti-TB drug-induced hepatotoxicity.^9,14,15^

 In the current study, none of the patients were treated with the simultaneous initiation of all four anti-TB drugs. Instead, 40.4% of patients were treated with the sequential initiation of drugs at full dosage, and 59.6% were treated with the sequential initiation of drugs at low dosage, gradually increasing to full dosage. The results of the study confirmed that the elimination of PZA from the re-administration regimen resulted in a shorter duration of hospitalization. It is important to note that PZA has been reported to cause more hepatotoxicity compared to other anti-TB drugs.^16,17^

 The American Thoracic Society recommends starting TB drug-induced liver treatment with full-dose RIF. If no signs of hepatotoxicity appear, INH is added in 3‒7 days. In severe cases, PZA may be omitted.^18^

 In contrast, the guidelines established by the British Thoracic Society recommend starting reintroduction treatment with INH, administered at a dose of 50 mg/d, which is gradually increased to 300 mg/d over 2‒3 days. If no adverse reactions are observed, RIF is added to the treatment regimen at a starting dose of 75 mg/d, which is increased to a range of 450‒600 mg/d, as appropriate for the patient’s weight, over 2‒3 days. Finally, PZA is added to the treatment regimen at a dose of 250 mg/d, which is gradually increased to a range of 1‒1.5 g per day over 2‒3 days.^19^

 In a pilot study conducted by Alpana et al, 32 TB patients were divided into three groups. Group 1 received INH and RIF on day 1, group 2 received RIF on day 1 and INH on day 8, and group 3 received INH on day 1 and RIF on day 8. The hepatotoxicity recurrence rate showed no significant difference among the three groups.^20^

 In our study, weight loss and HCV infection were identified as risk factors for the recurrence of hepatotoxicity. HBV infection was identified as a risk factor for longer hospitalization. Patients who did not have cardiovascular disease had a longer hospital stay due to having more renal and pulmonary disease. Additionally, the study found that re-challenge was more successful in male patients, and female gender was found to be a risk factor for recovery from hepatotoxicity. This is likely due to the higher activity of cytochrome CYP3A in women, which can increase the concentration of RIF and its metabolites in the blood, potentially leading to toxicity.^21,22^

 The present descriptive study focused on drug-induced hepatotoxicity caused by anti-TB drugs, risk factors, and rechallenge methods. Although this cross-sectional study can provide an estimate of the prevalence of drug-induced hepatotoxicity, it is considered weaker than other types of studies for establishing causality relationships between variables.^23^ Due to the high prevalence of TB and drug-induced hepatotoxicity in Iran, there are many questions in this regard, and conducting research in this area could be beneficial and valuable.

## Conclusion

 Drug-induced hepatotoxicity is a common occurrence that often results in treatment discontinuation. Understanding the prevalence of this complication and identifying appropriate methods of rechallenge treatment are crucial in reducing complications and mortality rates. In this study, weight loss and HCV infection were identified as risk factors for the recurrence of hepatotoxicity. Underlying heart disease and HBV infection were identified as risk factors for longer hospitalization. Additionally, the study results revealed that re-challenge was more successful in male patients, and female gender was a risk factor for recovery from hepatotoxicity. In addition, both methods of drug reintroduction—sequential at full dosage or gradual dosage increase—yield similar treatment outcomes.
